# Morphological and Physiological Responses of Cotton (*Gossypium hirsutum* L.) Plants to Salinity

**DOI:** 10.1371/journal.pone.0112807

**Published:** 2014-11-12

**Authors:** Lei Zhang, Huijuan Ma, Tingting Chen, Jun Pen, Shuxun Yu, Xinhua Zhao

**Affiliations:** State Key Laboratory of Cotton Biology, Institute of Cotton Research of CAAS, Anyang, P. R. China; Universidade Federal de Vicosa, Brazil

## Abstract

Salinization usually plays a primary role in soil degradation, which consequently reduces agricultural productivity. In this study, the effects of salinity on growth parameters, ion, chlorophyll, and proline content, photosynthesis, antioxidant enzyme activities, and lipid peroxidation of two cotton cultivars, [CCRI-79 (salt tolerant) and Simian 3 (salt sensitive)], were evaluated. Salinity was investigated at 0 mM, 80 mM, 160 mM, and 240 mM NaCl for 7 days. Salinity induced morphological and physiological changes, including a reduction in the dry weight of leaves and roots, root length, root volume, average root diameter, chlorophyll and proline contents, net photosynthesis and stomatal conductance. In addition, salinity caused ion imbalance in plants as shown by higher Na^+^ and Cl^−^ contents and lower K^+^, Ca^2+^, and Mg^2+^ concentrations. Ion imbalance was more pronounced in CCRI-79 than in Simian3. In the leaves and roots of the salt-tolerant cultivar CCRI-79, increasing levels of salinity increased the activities of superoxide dismutase (SOD), ascorbate peroxidase (APX), and glutathione reductase (GR), but reduced catalase (CAT) activity. The activities of SOD, CAT, APX, and GR in the leaves and roots of CCRI-79 were higher than those in Simian 3. CAT and APX showed the greatest H_2_O_2_ scavenging activity in both leaves and roots. Moreover, CAT and APX activities in conjunction with SOD seem to play an essential protective role in the scavenging process. These results indicate that CCRI-79 has a more effective protection mechanism and mitigated oxidative stress and lipid peroxidation by maintaining higher antioxidant activities than those in Simian 3. Overall, the chlorophyll a, chlorophyll b, and Chl (a+b) contents, net photosynthetic rate and stomatal conductance, SOD, CAT, APX, and GR activities showed the most significant variation between the two cotton cultivars.

## Introduction

The proportion of agricultural land that is negatively affected by high salinity is increasing worldwide, owing to natural causes and agricultural practices [1]. This problem has been aggravated by the development of more recent agricultural practices such as irrigation. Approximately 20% of the world's cultivated lands and more than half of all irrigated lands are affected by salinity [2]. High salt concentrations in the soil cause various events that negatively impact agricultural production, such as delays in plant growth and development, inhibition of enzymatic activities and reductions in photosynthetic rates [3]. Therefore, before attempts can be made to introduce genetic and environmental factors to alleviated salt stress, it is critical to elucidate the morphological and physiological responses of particular crops and cultivars to salinity.

In general, salt stress causes an imbalance of the cellular ions resulting in ion toxicity, osmotic stress and production of reactive oxygen species (ROS) [4], thus affecting plant growth, morphology, and survival [5]. High concentrations of NaCl outside the roots reduce the water potential and make it more difficult for the root to extract water. On the other hand, high concentrations of Na^+^ and Cl^−^ ions inside plant cells are inhibitory to many enzyme processes. Salt-tolerant plants can not only regulate ion and water movements more efficiently but should also have a better antioxidant system for effective removal of ROS [6]. Salt stress causes excessive generation of ROS such as superoxide anions (O_2_^−^), hydrogen peroxide (H_2_O_2_), and hydroxyl radicals (OH•) [7]. To mitigate the oxidative damage initiated by ROS formed under salt stress, plants possess a complex antioxidant system, including non-enzymatic antioxidants such as ascorbic acid, glutathione (GSH), tocopherols, and carotenoids; antioxidant enzymes such as superoxide dismutase (SOD, EC 1.15.1.1), catalase (CAT, EC 1.11.1.6), glutathione peroxidase (EC 1.11.1.9), and peroxidases (POD, EC 1.17.1.7); and enzymes of the so-called ascorbate-glutathione cycle, including ascorbate peroxidase (APX, EC 1.11.1.11) and glutathione reductase (GR, EC 1.6.4.2). These components of the antioxidant system act in concert to alleviate the cellular damage accumulated under conditions of oxidative stress [8], [9].

SOD is generally considered as the first line of the antioxidant defense system, as it catalyzes the dismutation of O_2_^−^ into H_2_O_2_ and O_2_ in the cytosol, chloroplasts, and mitochondria [10]. POD is mainly located in the apoplastic space and vacuoles, where it plays an important role in catalyzing the conversion of H_2_O_2_ to H_2_O and O_2_ [11]. H_2_O_2_ is scavenged by CAT and APX. CAT dismutates H_2_O_2_ to H_2_O and O_2_, whereas APX, together with monodehydroascorbate reductase, dehydroascorbate reductase, and GR, converts H_2_O_2_ to H_2_O via the ascorbate-glutathione pathway. APX is the first enzyme in this pathway, which eliminates H_2_O_2_ by using ascorbate as an electron donor in an oxidation-reduction reaction [12]. GSH is the final enzyme in this pathway and functions to protect plants from oxidative stress by maintaining GSH in the reduced state [13]. Under salt stress, malondialdehyde (MDA), the decomposition product of the polyunsaturated fatty acids of biomembranes, tends to accumulate [14]. Accordingly, cell membrane stability has been widely used to differentiate between stress-tolerant and -susceptible cultivars of some crops [15], in some cases, higher membrane stability could be correlated with abiotic stress tolerance.

In most plants, higher levels of the activity of the above-mentioned antioxidant enzyme are considered as a salt tolerance mechanisms [9]. Indeed, previous studies have shown that within the same species, salt-tolerant cultivars generally have enhanced or higher constitutive antioxidant enzyme activity under salt stress when compared with sensitive-cultivars. This has been demonstrated in numerous plant species such as cotton [16], rice [17], and pea [18]. Moreover, the response of plant antioxidant enzymes to salinity has been shown to vary among plant species, tissues, and subcellular localizations [19]. Several studies have demonstrated that salt-tolerant species show increased antioxidant enzyme activities and antioxidant contents in response to salt stress, whereas salt-sensitive species fail to do so [20]. Thus, the evidence accumulated to data indicates that intrinsic antioxidant resistance mechanisms of plants may provide a strategy to enhance salt tolerance. However, to achieve efficient selection of genetically-transformed salt-tolerant plants, the mechanisms underlying the effects of salt on the morphology, physiology, growth, and antioxidative responses of plants must first be identified [21].

Salt may affect plant growth indirectly by decreasing the rate of photosynthesis. Indeed, under saline conditions, substantial reduction in photosynthesis has been associated with a decrease in total chlorophyll content and distortion in chlorophyll ultra-structures [22]. Although the factors that limit photosynthesis in salt-stressed plants have been investigated for a number of species, the mechanistic pattern of inhibition remains unclear [23].

In addition, the relationships between ion accumulation, morphological and physiological changes, salt stress, and resulting plant injury are poorly understood. A number of conflicting views have been proposed in the literature over the toxic ions and enzymatic protection and activity involved in the response to salinity [24], [25]. Oxygen radicals are generated during plant metabolism that need to be scavenged by antioxidant systems to maintain normal growth; therefore, determining any adverse effects on these antioxidant systems due to salinity is an important consideration for appropriate cultivar selection.

Cotton is one of the most economically important crops in China. Although it is classified as a salt-tolerant crop, this tolerance is not only limited but also varies according to the growth and developmental stages of the plant [26]. Several studies have been conducted to assess the effect of salinity on the germination, vegetative growth, or yield of cotton [27]–[29]. However, the interactions between growths rates, ionic content, enzymatic activity, and oxidation reactions are likely to be complex and perhaps vary significantly between cultivars; therefore, such interactions deserve more detailed investigation.

In this study, the effect of NaCl on the growth behavior of two cotton cultivars that differ in their tolerance to salt was investigated. Changes in growth, ion concentration, pigment contents, and photosynthesis were assessed and linked to differences in the antioxidant system observed during salt stress. Since no detailed investigations have been conducted on this responses to data, the information presented here will not only provide criteria for improving salt-tolerant cotton specifically but also for selecting other salt-tolerant species and cultivars.

## Materials and Methods

### Experimental design

Seeds of two cotton cultivars, CCRI-79 (salt tolerant) and Simian 3 (salt sensitive), were obtained from the National Medium-Term Gene Bank of Cotton in China and soaked in sterile deionized water at 28°C for 6 h. They were then transferred to two sheets of sterile filter paper moistened with deionized water and placed in plastic trays for germination at 28°C for 72 h in the dark. The seeds were then sown into pots filled with perlite and grown under controlled conditions (light/dark regime of 16/8 h at 23°C, relative humidity of 60%–70%, photosynthetic photon flux density of 350 µmol⋅m^−2^⋅s^−1^). Germinated seeds were sown into holes in Styrofoam boards that were placed in deionized water and grown hydroponically in a growth room for 3 weeks under fluorescent and incandescent lights.

After 3 weeks, healthy and uniform seedlings were transplanted to 4-L plastic pots (10 plants per pot) filled with an aerated Hoagland nutrition solution (pH 5.2). The nutrition solution was aerated constantly and replaced twice a week throughout the experiment. Plants were cultured under non-saline conditions for 10 d to ensure full establishment before starting the salinity treatments. Salt stress treatment was initiated by providing the plants with full-strength Hoagland's solutions containing 0, 80, 160, or 240 mM NaCl. To avoid osmotic shock, salt concentrations were increased daily by 40 mM NaCl, until reaching the required concentration. After 1 week, the plants were harvested, cleaned and their fresh weights were measured.

### Leaf photosynthesis measurements

Net photosynthetic rate (Pn) and stomatal conductance (gs) of leaves were measured in three plants per cultivar per treatment with a photosynthesis system (Li-6400, Li-COR Inc., NE, USA) under 1500 µmol⋅m^2^⋅s^2^ light intensity, 65%±5% relative humidity, 32°C±2°C leaf temperature, and 380 µmol⋅mol^−1^ CO_2_ concentrations at 9:30–11:00 AM.

### Growth parameter measurements

From each treatment group, 10 plants were randomly selected and separated into leaf and root fractions. The leaf area of the youngest fully developed leaf of each plant was measured. Root samples were placed in a rectangular glass dish with a thin layer of water (4–5 mm depth) to allow all roots to spread appropriately. Entire roots were scanned with an EPSON Transparency unit (Epson Perfection V700 Photo; Indonesia), and then analyzed with WinRHIZO software version 5.0 (Regent Instruments, Inc.; Canada) to calculate the total root length, total root surface area, total root volume, and average root diameter. Leaves and roots were washed with deionized water and dried at 70°C for 48 h to determine dry weight before being ground to determine the ion contents.

In addition, another six plants per replicate of each of the four treatments were harvested. Fresh roots of seedlings were separated and frozen immediately in liquid nitrogen before being stored at −80°C pending further analysis.

### Chlorophyll and carotenoid content measurements

To determine chlorophyll a (Chl a), chlorophyll b (Chl b), and carotenoids levles, 3–5 discs (0.8-cm diameter) were cut from the upper-most fully expanded leaves randomly selected from five plants per replicate. Discs were homogenized with 2 mL of acetone (80%) and washed twice with an additional 2 mL of acetone. The absorbance of the pooled extracts was measured using a spectrophotometer at 480 nm, 645 nm, and 663 nm. Contents of Chl a, Chl b, and carotenoids in the extracts was determined using MacKinney equations [30]:
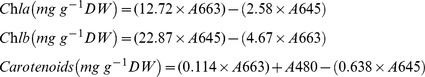



### Ion analyses

The concentration of calcium (Ca), magnesium (Mg), potassium (K), and sodium (Na) were analyzed in subsamples of dried plant materials, which were finely ground in a mill grinder. Approximately 0.5 g of finely ground plant samples were placed into digesting tubes, to which 10 mL of concentrated nitric acid and 3 mL perchlorate acid were added. All the samples were soaked for 12 h and then burned at 300°C for 3 h. The residue was transferred to a 50-mL volumetric flask, which was topped up to 50 mL with distilled water. The cation content was then measured using an atomic absorption spectrophotometer (TAS-986; Persee; China) [31]. For the determination of Cl^−^ content, leaf samples (0.1 g) were extracted in 10 mL of distilled water by heating at 80°C for 3 h [32]. The Cl^−^ content in the extracts was analyzed by ion chromatography (DX-300; Sunnyvale, CA, USA) [33].

### Determination of lipid peroxidation

Frozen leaf and root segments (0.5 g) were homogenized in a 0.1% (w/v) trichloroacetic acid (TCA) solution. The homogenate was centrifuged at 15,000× *g* for 10 min and 1 mL of the supernatant was added to 4 mL 0.5% (w/v) 2-Thiobarbituric acid (TBA) in 20% (w/v) TCA. The mixture was incubated at 90°C for 30 min, and the reaction was stopped by placing the reaction tubes in an ice water bath. Samples were centrifuged at 10,000× *g* for 5 min and the absorbance of the supernatant was read at 532 nm. The value for non-specific absorption at 600 nm was subtracted from the measured values. The concentration of MDA was calculated using an extinction coefficient of 155 mM^−1^·cm^−1^.

### H_2_O_2_ determination

H_2_O_2_ content was estimated according to the methods of Bernt and Bergmeyer [34]. Approximately 0.5 g of root and leaf samples from control and treatment groups were homogenized with liquid nitrogen, and the powders were suspended in 1.5 mL of 100 mM potassium phosphate buffer (pH 6.8). The suspensions were then centrifuged at 18,000× *g* for 20 min at 4°C. The enzymatic reaction was initiated with 0.25 mL supernatant and 1.25 mL peroxidase reagent, consisting of 83 mM potassium phosphate buffer (pH 7.0), 0.005% (w/v) *O*-dianizidine, and 40 µg peroxidase/mL at 30°C. The reaction was stopped after 10 min by adding 0.25 mL of 1 N perchloric acid and the reaction mixture was centrifuged at 5000× *g* for 5 min. The absorbance of the supernatant was read at 436 nm, and the amount of H_2_O_2_ was determined using an extinction coefficient of 39.4 mM^−1^⋅cm^−1^.

### Extraction and assay of antioxidant enzyme assay

For enzyme extractions, frozen root and leaf samples (0.3 g) were ground into a fine powder by using a mortar that was placed in an ice bath and a pestle that was pre-cooled with liquid nitrogen, and homogenized in 50 mM potassium phosphate buffer (pH 7.8) containing 1 mM ascorbate and 2% (w/v) polyvinylpolypyrrolidone. Homogenates were then centrifuged at 20,000× *g* for 30 min at 4°C.

SOD activity was determined according to the methods of Foster and Hess [35]. The reaction was performed in a total volume of 1 mL containing 50 mM potassium phosphate buffer (pH 7.8, containing 0.1 mM ethylenediaminetetraacetic acid [EDTA]), 0.1 mM cytochrome c, 0.1 mM xanthine, enzyme extract, and 0.3 U/mL of xanthine oxidase. The reaction was initiated by the addition of xanthine oxidase and absorbance was measured at 560 nm. One unit of SOD activity was defined as the amount of enzyme that inhibits the rate of cytochrome c reduction by 50%.

Total CAT activity was measured according to the method reported by Beers and Sizer [36], with minor modifications. The reaction mixture (1.5 mL) consisted of 100 mM phosphate buffer (pH 7.0), 0.1 mM EDTA, 20 mM H_2_O_2_, and 50 µL enzyme extract. The reaction was initiated by the addition of the enzyme extract. The decrease in H_2_O_2_ was monitored at 240 nm and was quantified by its molar extinction coefficient (36 M^−1^⋅cm^−1^).

Peroxidase activity was analyzed in 2.9 mL of 0.05 M phosphate buffer, containing 1.0 mL of 0.05 M guaiacol and 1.0 mL of 2% H_2_O_2_ [37]. Increases in absorbance at 470 nm were recorded after adding 2.0 mL of 20% chloroacetic acid.

APX activity was determined according to Nakano and Asada [38] by following the decline in absorbance at 290 nm as ascorbate was oxidized. The oxidation rate of ascorbate was estimated between 1 and 60 s after starting the reaction with the addition of H_2_O_2_. The 1-mL reaction mixture contained 50 mM HEPES-NaOH (pH 7.6), 0.22 mM ascorbate, 1.0 mM H_2_O_2_, and an enzyme extract. Corrections were made for the low, non-enzymatic oxidation of ascorbate in the absence of H_2_O_2_.

GR activity was measured as described by Foyer et al. [39]. The assay medium contained 1 mM EDTA in 50 mM potassium phosphate buffer (pH 7.8), 0.1 mM NADPH, enzyme extract, and 0.1 mM glutathiol (GSSG) in a total volume of 1 mL. The reaction was initiated by adding GSSG and the NADPH oxidation rate was monitored at 340 nm. GR activity was calculated using an extinction coefficient of 6.2 mM^−1^·cm^−1^ for NADPH, and one unit of enzyme was defined as the amount of enzyme required to oxidize 1 µmol NADPH per minute. The specific enzyme activity for all enzymes was expressed as units/mg protein.

### Statistical analysis

The experiments were set up as a completely randomized design, including two cotton cultivars and four salinity levels. All data obtained were subjected to ANOVA, and the mean difference was compared by the LSD test at 95% or 99% levels of probability. In all figures, the spread of values is shown as error bars representing standard errors of the means.

## Results and Discussion

### Growth parameters

**Figure 1:**
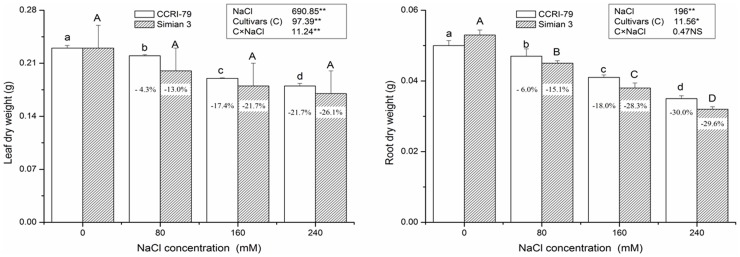
Effect of NaCl salinity on the leaf and root dry weight of two cotton cultivars. Vertical bars represent ± standard error (n = 3). Bars labeled with the different lowercase letters on open square bars or uppercase letters on closed square bars are significant difference (P<0.05). *, ** Significant at P<0.05 and P<0.01, respectively. NS, not significant. Figures in column indicate± increase/decrease under salinity stress over control.

Salinity exposure can lead to various physiological and biochemical changes within plant cells causing numerous changes in their structure and function [40]. In the present study, salt-induced changes in the growth and antioxidant profile of cotton plants were evaluated. Increasing NaCl concentration, up to 240 mM, gradually reduced leaf and root growth (Fig. 1). In general, both cultivars showed decreased growth rates of the roots and leaves with increasing salt concentration, but there was no significant variation in the leaf dry weight of Simian 3 when subjected to salt stress. However, the percentage reduction in leaf and root dry weights due to salinity over control was lower in CCRI-79 as compared with Simian 3, indicating that CCRI-79 is a more salt-tolerant cultivar. Inhibition of growth due to NaCl stress in CCRI-79 is comparable to the observations of Takemura et al. [41]. This reduction in growth may be due to osmotic injury or specific ion toxicity caused by the uptake of salt [42]. However, the differential response of growth to salinity observed between CCRI-79 and Simian 3 could be due to genotypic differences, which have also been reported by Qidar and Shams [26]. An increase in the tissue maintenance process (through respiration) is believed to be the primary cause of growth decline during salinity stress, and could represent a mechanism of adaptation to salinity [25]. The sacrifice of leaf photosynthetic tissue during salt adaptation may serve to conserve energy that can then be redirected to maintaining leaf multiplication and growth, indicating successful use of the tissue maintenance process. Thus, the fact that the leaf dry weight of the salt-sensitive cultivar Simian 3 was maintained indicates that it is not reallocating its energy reserves when faced with high-salinity conditions as compared to the more salt-tolerant cultivar CCRI-79.

When plants are grown under conditions of salt stress, the immediate response is a cessation in the expansion of the leaf surface [43]. Several authors have reported the phenomenon of leaf expansion in response to salinity in halophytes as well as in glycophytes [44]. Similarly, in our study, leaf area was highest in the control group (0 mM NaCl), whereas it decreased continuously as salinity increased (Table 1). One possible reason for this decline might be related to salt osmotic effects, which affect cell turgor and expansion [45].

**Table 1:** Morphological parameters of cotton at different NaCl concentrations and results of ANOVA (F-ratios).

Cultivar	NaCl (mM)	Leaf area (cm^2^)	Root length (cm)	Surface area (cm^2^)	Volume (cm^3^)	Average diameter (mm)
CCRI-79	0	36.19±0.91a	370.86±3.45a	52.07±0.94a	0.57±0.014a	0.54±0.016a
	80	35.18±1.06a	320.33±2.53b	44.04±1.29b	0.48±0.016b	0.45±0.014b
	160	34.43±0.46a	192.72±4.38c	32.45±1.67c	0.42±0.009c	0.43±0.009b
	240	34.07±0.12a	161.28±4.88d	28.03±0.71d	0.34±0.014d	0.35±0.007c
Simian 3	0	35.13±0.85a	302.58±3.02a	49.07±0.78a	0.62±0.041a	0.52±0.005a
	80	33.99±1.07a	260.86±8.67b	35.71±1.28b	0.39±0.043b	0.49±0.018a
	160	33.46±1.36a	188.32±14.26c	31.12±0.98c	0.38±0.023b	0.40±0.014b
	240	32.98±0.63a	138.58±1.05d	24.19±0.60d	0.30±0.008c	0.31±0.004c
NaCl		4.31NS	673.99**	338.36**	75.49**	182.17**
Cultivars(C)		5.73*	134.92**	50.99**	4.60 NS	3.12 NS
C×NaCl		0.01 NS	20.54**	6.70*	5.15*	9.41**

Values are the mean of three replicates ± S.E. Means followed by a different letter within a column for each cotton cultivar are significantly different at P<0.05 according to the Student's LSD test.

*, ** Significant at P<0.05 and P<0.01, respectively.

NS, not significant.

Compared with the no-salt control treatment, salt stress significantly (P<0.01) reduced the length, surface area, volume, and average diameter of the roots in both cotton cultivars (Table 1). Significant differences were observed when NaCl concentration was increased from 80 to 240 mM. There are several reasons for the reduced root length, including cell growth restriction due to low water potential of the external medium, interference caused by ions, or the toxicity of accumulated ions [46]. Our findings are consistent with the results of Siroka et al. [47], who reported that salinity suppressed the development of maize roots cell. The inhibition of root growth in terms of root length, surface area, volume, and average diameter can be attributed to the inhibition of mitosis, reduced synthesis of cell wall components, damage to the Golgi apparatus, and changes in polysaccharide metabolism [48]. However, this decrease was more predominant in Simian 3 than in CCRI-79, indicating that CCRI-79 was more tolerant to salinity than Simian 3.

### Chlorophyll and carotenoid contents and photosynthesis

**Figure 2:**
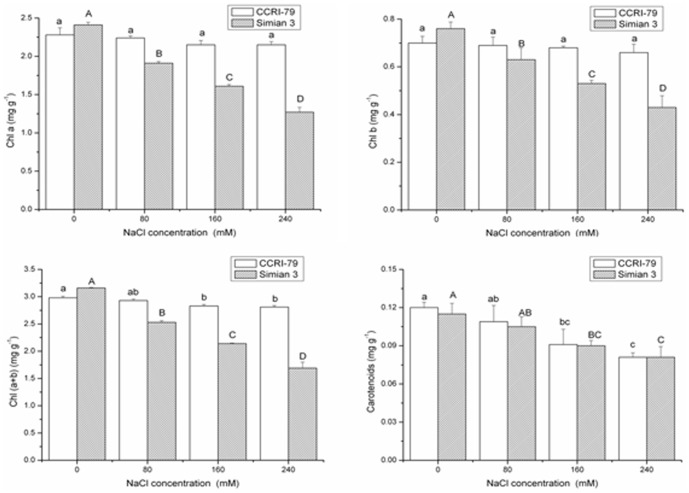
Concentrations of chlorophylls and carotenoids in cotton grown at different NaCl concentrations. Vertical bars represent ± standard error (n = 3). Bars labeled with the different lowercase letters on open square bars or uppercase letters on closed square bars are significant difference (P<0.05).

In general, the reduction in growth and productivity when plants are grown under conditions of salt stress is accompanied by as strong reduction in the rate of photosynthesis owing to severe impairments in photosynthetic activities and the photosynthetic apparatus, the degree of which depends on the varieties of species considered [49]. As shown in Fig. 2, the contents of Chl a, Chl b, and Chl (a+b) in the plants significantly (P<0.01) decreased as salinity increased in Simian 3, but only marginal changes were observed in CCRI-79 (Table 2). Maintenance of chlorophyll content has been reported in other salt-tolerant crops such as durum wheat and legume species [50]. One possible mechanisms of salt tolerance in these species may be the possession of a salt exclusion and/or sequestration trait that prevents leaf injury, thus maintaining chlorophyll content. Since chlorophyll content is directly correlated with growth and development of the plant [51], the decrease in chlorophyll content in Simian 3 suggested substantial damage to the photosynthetic mechanism, as reported previously in salt-treated rice and sorghum plants [52], [53]. The inhibitory effects of salt on chlorophylls could be due to the suppression of specific enzymes responsible for the synthesis of chlorophyll. Our findings regarding total chlorophyll content are comparable with the observations of Meloni et al. [54], who demonstrated that Guazuncho, another salt-sensitive cotton cultivar, showed a 35% reduction in total chlorophyll content after 21 days of salinity treatment.

**Table 2:** *F* values of ANOVA of the effects of NaCl, cultivars, and their interaction for chlorophylls, carotenoids content, net photosynthetic rate (Pn), and stomatal conductance (gs).

Item	NaCl	Cultivars(C)	C×NaCl
Chl a	108.52**	245.65**	66.29**
Chl b	45.49**	61.62**	26.86**
Chl (a+b)	186.62**	380.12**	113.08**
Carotenoids	13.62**	0.28 NS	0.10 NS
Pn	98.15**	200.15**	51.5**
gs	56.31**	77.17**	23.44**

*, ** Significant at P<0.05 and P<0.01, respectively.

NS, not significant.

Carotenoids are reported to play an important role in ROS scavenging, thereby protecting membranes from salt stress [55]. However, we did not observe changes in carotenoid contents in response to salinity treatments in either cultivar, suggesting that these pigments are not involved in the response to salt stress in cotton. Although the rate of change was slower in carotenoids than in chlorophylls, carotenoid content also showed a decreasing trend with increasing salt stress, indicating that this trait could also serve as a useful indicator of NaCl stress in cotton.

**Figure 3:**
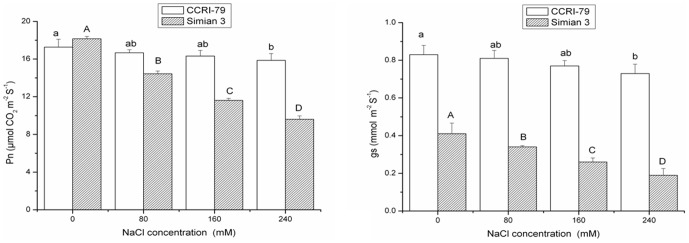
Net photosynthetic rate (Pn) and stomatal conductance (gs) of two cotton cultivars as affected by different NaCl concentrations. Vertical bars represent ± standard error (n = 3). Bars labeled with the different lowercase letters on open square bars or uppercase letters on closed square bars are significant difference (P<0.05).

Since plant growth is dependent on photosynthesis, environmental stresses affecting growth will also affect photosynthesis [56]. In the present study, the net photosynthetic rate (Pn) and stomatal conductance (gs) of both cotton cultivars were inhibited by salinity due to NaCl (Fig. 3, Table 2). However, the net photosynthetic rate and stomatal conductance were significantly lower for Simian 3 than for CCRI-79 under conditions of salt stress. Compared with the control treatment, the net photosynthetic rate and stomatal conductance of Simian 3 significantly decreased with increasing salinity, whereas there was no significant difference in either trait in CCRI-79.

Photosynthesis was reduced in both cotton cultivars in response to salt stress, which was likely caused by the reduction in stomatal conductance. Indeed, parallel decreases in stomatal conductance and net photosynthesis due to NaCl salinity have previously been reported for cotton [57]. Our results suggest that stomatal closure limited the leaves' photosynthetic capacity in the NaCl-treated plants of both cultivars, Although only Simian 3 showed a significant decline in the leaf chlorophyll contents due to NaCl stress for 7 days. Similarly, Delfine et al. reported no changes in the chlorophyll content in spinach plants (*Spinacia oleracea* L.) exposed to salt stress for 20 days [58]. Our results suggest that the greater reduction in stomatal conductance accompanied by decreased leaf chlorophyll content could have contributed to the higher reduction in the leaf photosynthetic rate of Simian 3 when compared with that of CCRI-79.

Further examination showed that the decrease in the Chl a, Chl b, Chl (a+b), net photosynthetic rate, and stomatal conductance levels with increasing salt concentrations occurred more rapidly compared with the rate of decrease in carotenoid content; this trend was more conspicuous in Simian 3 than in CCRI-79.

### Ion concentrations

**Figure 4:**
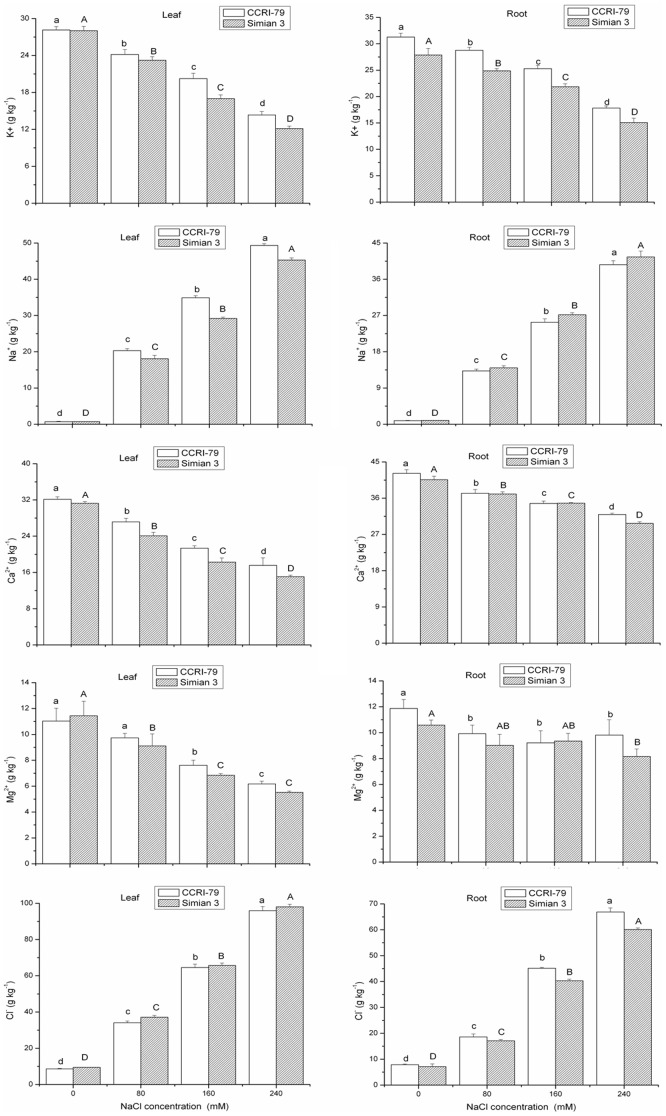
Effect of NaCl salinity on the concentrations of K^+^, Na^+^, Ca^2+^, Mg^2+^ and Cl^−^ in the roots and leaves of cotton. Vertical bars represent ± standard error (n = 3). Bars labeled with the different lowercase letters on open square bars or uppercase letters on closed square bars are significant difference (P<0.05).

High external salt concentration causes an ion imbalance or disturbance in ion homeostasis [43]. In our experiments, the leaves and roots of both cultivars had higher levels of Na^+^ and Cl^−^ ions under salt stress due to nonspecific ion uptake and/or membrane leakage. However, as the NaCl concentration increased, the levels of Na^+^ and Cl^−^ also increased further, suggesting that these cultivars may not differ in terms of Na^+^ uptake and its transportation to leaves, and thus the increase observed can not be explained by an ionic exclusion mechanism (Fig. 4). In addition, Na^+^ ion concentrations in leaves were higher than those in the roots of both cultivars at all salinity levels, which indicated the inability of these cultivars to prevent Na^+^ ion transportation from the roots to leaves. Chlorine ions showed a similar distribution pattern to Na^+^ ions, despite being at higher salinity levels. Na^+^ and Cl^−^ are highly water-soluble and are readily taken up by plants and transported into leaves; these ions most likely act as osmotica, but only moderate concentrations can be tolerated before growth and photosynthesis are reduced.

High Na^+^ and Cl^−^ absorption competes with the uptake of other nutrient ions such as K^+^, Mg^2+^, and Ca^2+^, leading to a deficiency of these ions and an imbalance among cations [59]. During the same period, K^+^ and Ca^2+^ content in the leaves and roots of both cultivars were significantly reduced, and a further decrease in these ions was observed with increasing NaCl concentration (Fig. 4, Table 3). Salt tolerance involves not only adaptation to Na^+^ influx but also acquisition of K^+^, the uptake of which is adversely affected by high external Na^+^ concentration due to the chemical similarity of these two ions [60], [61]. Indeed, selective uptake of K^+^ as opposed to Na^+^ is considered to be one of the key physiological mechanisms contributing to salt tolerance in many plant species [62]. K^+^ efflux has already been used as an indicator of cellular toxicity for a range of toxic compounds, and losses of K^+^ and Ca^2+^ have already been documented during salinity stress [63], [64]. A large and permanent efflux of K^+^ and Ca^2+^ usually indicates damage to the limiting membranes.

**Table 3:** *F* values of ANOVA of the effect of NaCl, cultivars, and their interaction for K^+^, Na^+^, Ca^2+^, Mg^2+^, and Cl^−^ contents.

Tissue	Item	Cultivars (C)	NaCl	C×NaCl
Leaf	K^+^	22.47**	349.67**	4.05 NS
	Na^+^	131.7**	5755.89**	22.16**
	Ca^2+^	27.27**	220.03**	1.28 NS
	Mg^2+^	2.09 NS	69.77**	0.94 NS
	Cl^−^	7.03*	3175.89**	0.58 NS
				
Root	K^+^	72.66**	204.40**	0.34 NS
	Na^+^	21.19**	4546.89**	3.12 NS
	Ca^2+^	15.30**	311.67**	4.96*
	Mg^2+^	5.19NS	6.21*	0.91 NS
	Cl^−^	65.95**	3497.79**	11.22**

*, ** Significant at P<0.05 and P<0.01, respectively.

NS, not significant.

On the other hand, salt treatments induced a significant decrease in Mg^2+^ concentrations in cotton leaves in the present study. The significantly lower levels of Mg^2+^ in the leaves under salinity conditions are probably related to the lower levels of chlorophylls present in the NaCl-treated leaves. However, in the roots of NaCl-treated plants, Mg^2+^ concentrations were close to or lower than those observed in control plants, even at the highest salt dose (240 mM NaCl). This was in contrast to the Ca^2+^ content, which was significantly lower in salt-treated plants. Our findings are similar to those of Khan [65], who reported that NaCl treatment induced a decline in Ca^2+^ and Mg^2+^ levels in *Ceriops tagal* plants.

In Addition, there were no significant differences between the two cotton cultivars in the variation of ion content (including K^+^, Na^+^, Mg^2+^, Ca^2+^ and Cl^−^) under NaCl stress. This similarity in ionic levels may be a consequence of the shoot culture method used, which would not involve a selective mechanisms of ion transport. For example, there was likely to be no regulation between the xylem parenchyma and xylem interface, in which ion selection and reabsorption from the medium may be regulated. Previous results reported for other species are consistent with these observations [66]. However, the restriction of entry of ions into metabolically active areas of cells in the more-tolerant CCRI-79 cultivar can not be ruled out as a mechanism to maintain ionic equilibrium when ions are highly concentrated in the external environment [67].

### Lipid peroxidation and proline content

**Figure 5:**
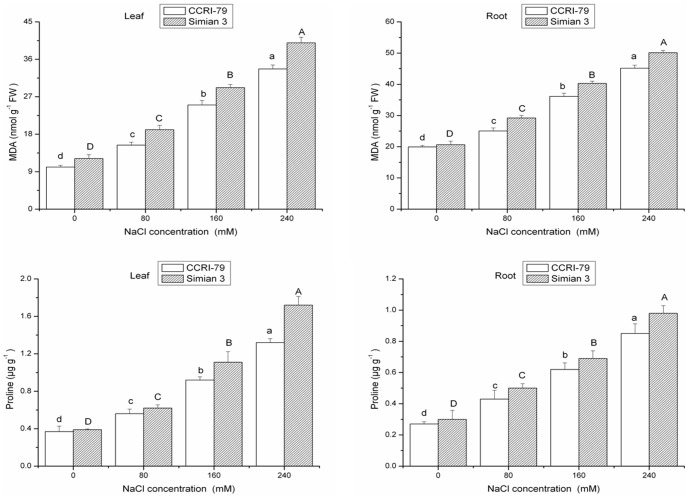
Effect of NaCl salinity on the concentrations of malondialdehyde (MDA) and proline in the roots and leaves of cotton. Vertical bars represent ± standard error (n = 3). Bars labeled with the different lowercase letters on open square bars or uppercase letters on closed square bars are significant difference (P<0.05).

Salt stress is known to result in extensive lipid peroxidation, which has often been used as an indicator of salt-induced oxidative damage in membranes [68]. The MDA content increased with increasing salinity in the leaves and roots of both cotton cultivars (Fig. 5, Table 4), indicating cell membrane damage in both cotton cultivars. However, as the salinity increased, the accumulation of MDA was higher in Simian 3 as compared to CCRI-79, indicating a higher degree of lipid peroxidation in Simian 3 due to salt stress. Lipid peroxidation could be a result of light-dependent formation of singlet oxygen during stress conditions [69]. The low values of MDA content obtained with CCRI-79 might account for the lower lipid peroxidation levels observed and the reduced effect on membrane permeability. Low levels of lipid peroxidation may have contribute to the observed tolerance of CCRI-79 plants exposed to high levels of salinity. Similar results for lipid peroxidation have been reported by other researchers in barley [70].

**Table 4:** *F* values of ANOVA of the effect of NaCl, cultivars, and their interaction for malondialdehyde (MDA), proline, H_2_O_2_ content, and antioxidant enzyme activities.

Tissue	Item	Cultivars (C)	NaCl	C×NaCl
Leaf	MDA	78.15**	608.62**	3.65 NS
	Proline	25.01**	230.23**	6.58*
	H_2_O_2_	74.86**	1357.74**	11.54**
	SOD	315.65**	213.58**	63.01**
	CAT	874.75**	445.12**	43.46**
	POD	0.47 NS	5.52*	0.49 NS
	APX	1.36 NS	5.93*	0.08 NS
	GR	28.88**	194.18**	15.77**
				
Root	MDA	60.16**	708.42**	4.47*
	Proline	9.10*	119.51**	0.72 NS
	H_2_O_2_	174.18**	4622.30**	40.92**
	SOD	8559.93**	1510.48**	1357.86**
	CAT	111.07**	1.92 NS	28.60**
	POD	50.42**	322.16**	2.08 NS
	APX	37.10**	208.76**	5.83*
	GR	17.47**	82.69**	1.45 NS

*, ** significant at P = 0.05 and P = 0.01 levels, respectively.

NS, not significant.

H_2_O_2_, hydrogen peroxide; SOD, superoxide dismutase; CAT, catalase; POD, peroxidase; APX, ascorbate peroxidase; GR, glutathione reductase.

Many plants accumulate proline as a nontoxic and protective osmoylte under saline conditions [55]. In this study, the levels of proline continued to increase in both of the cultivars as the NaCl concentration increased (Fig. 5). The proline concentration of CCRI-79 plants was lower than that of Simian 3 plants, especially at the highest salinity level, which could be attributable to the greater salt resistance of the CCRI-79 cultivar, i.e., less injury was induced by the salt [71]. Similar trends were observed by Rabie and Almadini in broad bean plants [72]. Moreover, in our study, proline accumulation in the leaves was higher than that in the roots, which is similar to the findings of Sharma and Dietz [73], indicating that proline plays a more important protective role in the leaves of cotton seedlings than in the roots under salinity stress.

### H_2_O_2_ content

**Figure 6:**
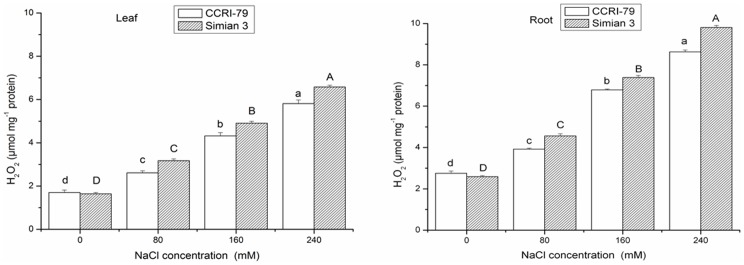
Effect of NaCl salinity on the concentrations of H_2_O_2_ in the roots and leaves of cotton. Vertical bars represent ± standard error (n = 3). Bars labeled with the different lowercase letters on open square bars or uppercase letters on closed square bars are significant difference (P<0.05).

Stress conditions enhance H_2_O_2_ production in different compartments of plants cells through both enzymatic and non-enzymatic processes [74]. In our study, both genotypes had similar levels of H_2_O_2_ levels at 0 mM NaCl treatment. However, salinity treatments caused a marked increase in H_2_O_2_ content, and the Simian 3 cultivar had a higher H_2_O_2_ content than did CCRI-79 after NaCl treatment (Fig. 6, Table 4), which could be mainly due to the decreased H_2_O_2_-scavenging activity in the salt-sensitive cultivar. These results are comparable to those reported in a previous study [75], where accumulation of H_2_O_2_ in the roots of the salt-tolerant rice cultivar FL478 was significantly higher than that of the salt-sensitive rice cultivar IR-29 in response to moderate salt stress applied for 12 days. Simultaneously, the H_2_O_2_ content were markedly lower in the leaves than in the roots, regardless of NaCl dose, which contrasts with the findings of Lee et al. [76] in rice. This discrepancy in the effects of salt on H_2_O_2_ content between studies may be related to technical difficulties, and therefore these results should be evaluated with caution [77]. Furthermore, H_2_O_2_ has been shown to induce cytosolic APX [78]; therefore, accumulation of H_2_O_2_ under high salinity conditions may be a signal to initiate an adaptive response to the stress [79]. Although differences in salt tolerance among different cultivars are not necessarily related to differences in the ability to detoxify ROS, many comparative studies using salt-tolerant and salt-sensitive genotypes have shown a correlation between salt tolerance and increased activity of antioxidant enzymes [80].

### Activities of antioxidant enzymes

Environmental stresses that limit photosynthesis can increase oxygen-induced cellular damage due to increased ROS generation [81]. Therefore, salt stress resistance may depend, at least in part, on the enhancement of the antioxidative defense system, which involves antioxidant compounds and several antioxidant enzymes. In the present study, the responses of SOD, POD, CAT, APX, and GR enzyme activities suggested that oxidative stress is an important component of salt stress in cotton plants.

**Figure 7:**
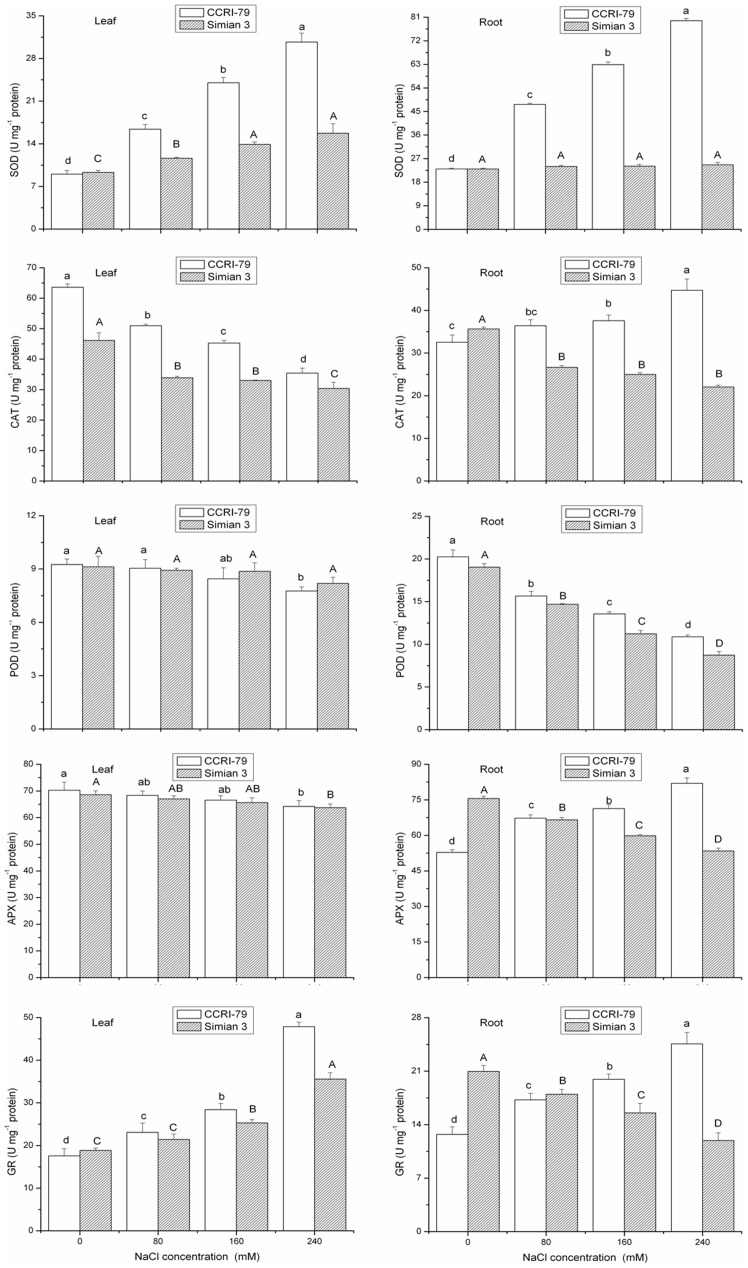
Effect of NaCl salinity on the antioxidant enzyme activities in the roots and leaves of cotton. Vertical bars represent ± standard error (n = 3). Bars labeled with the different lowercase letters on open square bars or uppercase letters on closed square bars are significant difference (P<0.05). SOD, superoxide dismutase; CAT, catalase; POD, peroxidase; APX, ascorbate peroxidase; GR, glutathione reductase.

Because SOD can catalyze the dismutation of superoxide to molecular oxygen and H_2_O_2_, this enzyme is considered the most effective intracellular enzymatic antioxidant. Indeed, it has been suggested that SOD plays an important role in plant stress tolerance and provides the first line of defense against the toxic effects of elevated levels of ROS [82]. In this study, salt stress increased SOD activity in the leaves of both cultivars and in the roots of CCRI-79 only (Fig. 7, Table 4). However, increased SOD activity in both the leaves and roots was more conspicuous in the salt tolerant cultivar CCRI-79 than in the salt-sensitive cultivar Simian 3, suggesting that the salt-tolerant genotype has a more efficient O_2_^•−^ radical-scavenging ability. Similar results have also been shown in both the leaves and roots of cotton and pea plants [14], [83]. In plants, high induction of SOD activity can lead to H_2_O_2_ accumulation as well as lipid peroxidation [84], which could contribute to the increased H_2_O_2_ content observed in the roots than in the leaves of cotton seedlings exposed to salinity.

POD is the primary enzyme that detoxifies H_2_O_2_ in the chloroplasts and cytosol of plant cells [85]. CAT plays an important role in the antioxidant system because it converts H_2_O_2_ into oxygen and water [86]. These two enzymes constitute the main H_2_O_2_-scavenging systems in cells. The present data showed that the roots had higher POD activity compared to the leaves in both cultivars; however, the enzyme activity in the roots and leaves responded differently to incremental levels of salinity. In the roots of both cultivars, there was a significant decline in POD activity with an increase in salinity levels, whereas there was no significant difference in the leaf of Simian 3 across salt treatments. However, POD activity in the leaf of CCRI-79 showed no significant difference when subjected to 0 to 160 mM NaCl concentrations. Conversely, the 240 mM NaCl concentration induced a significant decrease in the leaf POD activity of CCRI-79 when compared to the control (0 mM NaCl concentration). Amor et al. reported that H_2_O_2_ accumulation under salinity stress was related to a decrease in CAT activity in *C. maritima* [87]. In our study, CAT activity in the leaves and roots of Simian 3 declined with an increase in NaCl concentration (Fig. 7, Table 4). However, although the CAT activity in CCRI-79 increased with salinity in the roots, it decreased with increasing salinity in the leaves. CAT activity in the leaves and roots of CCRI-79 was significantly higher than that in Simian 3 leaves and roots for all salinity treatments. This indicates that the scavenging of H_2_O_2_ is more effective in CCRI-79 than in Simian 3. The roots of both the CCRI-79 and Simian 3 cultivars showed higher CAT activities than did the leaves. The stimulation of POD and CAT suggests that these enzymes are important in the detoxification of H_2_O_2_ in plant seedlings under salinity stress [88]. In contrast, POD and CAT activities were found to be reduce in response to excess salinity in the leaves and roots of various other plants species [52], [89]. Thus, the effect of salinity on antioxidant enzyme activities varies among plant species, organs, and even treatment concentrations.

H_2_O_2_ scavenging is also accomplished by the glutathione-ascorbate cycle, a series of coupled redox reactions involving three enzymes, APX, GR and monodehydroascorbate reductase [90]. APX plays a key role in protecting the plant against oxidative stress by scavenging H_2_O_2_ in different cell compartments. It also has a higher affinity for H_2_O_2_ than POD and CAT and, as such, may play a more crucial role in the management of ROS during stress [82]. As shown in Fig. 7, there was a significant difference in the effect of salt on APX activity between the leaves and roots of the two cultivars. As salinity increased, APX activity in the root increased in CCRI-79 but decreased in Simian 3. However, there was no significant difference in the leaf APX activity of plants subjected to 0 to 160 mM NaCl concentrations in both cultivars. Conversely, the 240 mM NaCl concentration induced a significant decrease in the leaf APX activity of both cultivars when compared to the control (0 mM NaCl concentration). We also found that higher APX activities were accompanied by higher CAT activities in CCRI-79, implying that CCRI-79 has a more effective H_2_O_2_-scavenging mechanism than Simian 3.

The role of GSH and GR in H_2_O_2_ scavenging in plant cells has been well established in the Halliwell-Asada enzyme pathway. GR catalyzes the rate-limiting and last step of the ascorbate-glutathione pathway [91]. In our study, GR activities increased in the leaves of both cotton cultivars and in the roots of CCRI-79 only in response to salt stress. On the other hand, salinity significantly reduced GR activity in the roots of Simian 3. Several studies investigating salt-tolerant and salt-sensitive cultivars have suggested that the salt tolerance trait is related to increased GR activity in salt-tolerant cultivars [83], [92], which is similar to our results. The elevated levels of GR activity may increase the rate of NADPH oxidation to NADP^+^, thereby ensuring the availability of NADP^+^ to accept electrons from the photosynthetic electron transport chain. Under such conditions, the flow of electrons to O_2_^−^, and therefore the formation of O_2_^−^ can be minimized. However, in the roots of Simian 3, the reduction in GR activity suggests a decrease in the GSH turnover rate. Considering that salinity also reduced APX activity in the roots of Simian 3, these results suggests that salt-sensitive cultivars exhibit a less-active ascorbate-glutathione cycle in the roots, which may be a key enzyme for the development of salt-tolerance in cotton plants.

Furthermore, the variation in SOD, CAT, APX, and GR activities differed significantly between CCRI-79 and Simian 3 under the NaCl concentrations tested. The increased salinity resistance of CCRI-79 was associated with its ability to maintain higher activity of these antioxidant enzymes, which resulted in lower H_2_O_2_ production, lipid peroxidation, and higher membrane stability. This provides further evidence that the H_2_O_2_-scavenging mechanisms were more effective in CCRI-79. By contrast, the relatively lower CAT, APX, and GR activities in salt-stressed Simian 3 compared to control plants indicated that H_2_O_2_ scavenging was less effective in this cultivar. This excess of H_2_O_2_ may be the main contributor to the extensive lipid peroxidation and growth inhibition observed in Simian 3.

When compared with the other scavenging enzymes tested, CAT and APX had much higher H_2_O_2_-scavenging activities in the leaves and roots of both cotton cultivars in our study. The importance of these two enzymes in H_2_O_2_ scavenging has been demonstrated in several previous studies, in which increased CAT and APX activities were correlated with tolerance to salt and other environmental stresses [93]. These enzymes were also shown to be important in salt tolerance of barley and mulberry [94], [95]. Therefore, the results suggest that the coordination of CAT and APX with SOD activity could comprise an additional constituent in the enzymatic antioxidant mechanism of cotton plants against oxidative stress.

## Conclusions

In this study, we compared the response of two cotton cultivars that differ with respect to salt tolerance to increasing NaCl concentrations. Overall, salinity significantly reduced the leaf and root dry weights, root volume, root length, root surface area, root average diameter, chlorophyll content and photosynthesis in the cotton plants of both cultivars. In contrast, antioxidant enzyme activity and proline and MDA contents increased in response to salinity. The salt-tolerant cultivar CCRI-79 showed evidence of possessing a more efficient antioxidant defense system against oxidative stress and lipid peroxidation by maintaining higher SOD, CAT, APX, and GR activities than those in Simian 3 during salt stress. The differences in the antioxidant enzyme activity of the leaves and roots may, at least in part, explain the greater tolerance to salt stress exhibited by CCRI-79 compared to that exhibited by Simian 3. Besides differences in antioxidant enzyme activities, the two cotton cultivars also showed marked variation in Chl a, Chl b, and Chl (a+b) contents, net photosynthetic rate, and stomatal conductance in response to NaCl stress. Therefore, acquisition of tolerance to salt may not only involve improved resistance to oxidative stress owing to enzymes that primarily function to protect membranes and tissues from such damage, but might also involve improvement in the biosynthetic pathway of photosynthetic pigments to maintain higher rates of photosynthesis in the face of stress. However, it should be noted that salt stress was only assessed at concentrations of 0, 80, 160, and 240 mM NaCl; therefore, further studies should be conducted to verify and screen the selection criteria for salt-tolerant species and cultivars. Nonetheless, the data presented herein provide novel information on the mechanisms and traits involved in salt tolerance, which could be exploited for cultivar selection and breeding to increase crop production in the face of increased salinity stress.
